# Concordant Oral-Genital HPV Infection in South Africa Couples: Evidence for Transmission

**DOI:** 10.3389/fonc.2013.00303

**Published:** 2013-12-12

**Authors:** Samantha L. Vogt, Patti E. Gravitt, Neil A. Martinson, Jennifer Hoffmann, Gypsyamber D’Souza

**Affiliations:** ^1^Department of Internal Medicine, University of Pittsburgh Medical Center, Pittsburgh, PA, USA; ^2^Department of Epidemiology, Johns Hopkins School of Public Health, Baltimore, MD, USA; ^3^Perinatal HIV Research Unit, University of Witwatersrand, Johannesburg, South Africa; ^4^Johns Hopkins University School of Medicine, Baltimore, MD, USA

**Keywords:** HIV, oral sex, South Africa, HPV, oral, genital, concordance, transmission

## Abstract

**Objective**: Cervical cancer is a leading cause of cancer mortality in South Africa. However, little is known about oral human papillomavirus (HPV) infection in high human immunodeficiency virus (HIV) seroprevalence settings.

**Method**: Thirty-four adult heterosexual couples attending an HIV testing center in Soweto, South Africa were enrolled. Each participant provided an oral rinse sample and genital swab, which were tested for 37 types of HPV DNA, and completed a risk behavior survey.

**Results**: Median age was 31 years and 9% (3/34) of men and 29% (10/34) of women enrolled tested HIV-positive; median CD4 count was 437 cells/mm^3^. Oral HPV prevalence was similar in women and men (12 vs. 18%, *p* = 0.48), and was non-significantly higher in HIV-infected vs. HIV-uninfected (23 vs. 13%, *p* = 0.34) subjects. Most men (82%) and women (84%) reported ever performing oral sex. Median number of lifetime sexual partners was “2–5” while median number of lifetime oral sex partners was 1. Oncogenic HPV subtypes were detected in 4% of oral, 26% of penile, and 74% of vaginal samples, including HPV16 in 1, 12, and 21% of these samples respectively. Genital HPV prevalence was significantly higher than oral HPV prevalence (75 vs. 15%, *p* ≤ 0.001). Thirty-five percent of couples (12/34) had at least one type-specific concordant vaginal-penile HPV infection but only one of nine couples with oral HPV had concordant oral–oral infection. However, 67% (4/6) of men and 25% (1/4) of women with oral HPV infection had partners with concordant genital HPV infection.

**Implications and Impact**: Oral–oral HPV concordance between couples is low, but oral-genital and genital–genital HPV concordance is higher, including concordance of male oral HPV infection with their partners’ vaginal HPV infection. This data is consistent with possible transmission of vaginal HPV infection to the oral cavity of sexual partners performing oral sex.

## Introduction

Human papillomavirus (HPV) is a sexually transmitted infection that causes squamous cervical cancer and some vaginal and anal cancers (1, 2). More recent data shows that HPV also causes a subset of head and neck cancers including 50–75% of oropharyngeal cancers (3). While initial cross-sectional studies from the United States suggest that oral HPV prevalence is elevated among human immunodeficiency virus (HIV)-infected individuals and further increased with immunosuppression (4, 5), there is currently limited information about oral HPV infections in Africa. Yet the incidence of oral cancer in South Africa is 2.7 per 100,000 per year and the incidence of pharyngeal cancer is 2.4 per 100,000 per year (6).

In South Africa, HIV prevalence is estimated at 17.3%, and 5.6 million people are living with HIV as of 2011 (7). Since the advent of effective antiretroviral therapy (ART) and its roll-out in Sub-Saharan Africa, HIV-infected patients are living longer. With this increase in survival comes an increased opportunity for progression of oncogenic viral infections into malignancies, including increased incidence of HPV-associated oral, genital, and anal cancers (8, 9).

Cervical HPV infection has been well studied in South Africa, where cervical cancer is one of the leading causes of cancer mortality in women (10). The prevalence of cervical HPV infection approaches 80% among HIV-infected women (11) in whom high rates of cervical premalignant lesions have been reported (12), an effect apparently mitigated by ART (13). Owing to limited data on oral HPV, the concordance of oral-cervical HPV infection has not been well studied. Previous cross-sectional data from the U.S. suggests that the risk factors such as tobacco use, immunosuppression, and increasing age might also be risk factors for persistent oral HPV infection (5, 14).

We therefore evaluated oral and genital HPV prevalence, risk factors and concordance among couples attending the Perinatal HIV Research Unit at Chris Baragwanath Hospital in Johannesburg, South Africa.

## Materials and Methods

### Study population

In 2011 we recruited 38 couples attending the Zazi Center at the Perinatal HIV Research Unit, Chris Baragwanath Hospital in Soweto, South Africa for HIV testing. The Zazi Center provides free, walk-in, voluntary HIV counseling and testing for individuals and couples. Clients who test positive for HIV receive CD4 count testing to assist in appropriate referral to care. To be included in this study, participants were required to be 18 years or older and be able to read English. A convenience sample that included all couples coming to the Zazi HIV testing clinic during the enrollment period were asked by their HIV testing counselor if they were interested in participating in the study. Roughly 33% of the individuals who came to the HIV clinic during this time period came without a partner and were therefore not eligible for this study. Sexually active couples who reported being together for at least 1 month were eligible for enrollment as long as both partners were willing to participate and provide informed consent. All 38 couples referred to the study coordinator enrolled in the study.

A couple was defined as two individuals (regardless of gender) who self-identified as current sexual partners. Exclusion criteria included an inability to provide consent, or an unwillingness to provide an oral rinse sample and survey data. Individuals could opt out of providing a genital sample. The study was approved by the Johns Hopkins Bloomberg School of Public Health institutional review board and the University of Witwatersrand human research ethics committee.

Each study subject provided an oral rinse and gargle sample, a genital swab and completed a risk behavior survey. Oral rinse and gargle specimens were obtained by having a participant gargle 10 ml of Scope mouthwash for a total of 30 s (10 s rinse, 5 s gargle, 10 s rinse, 5 s gargle). Specimens were collected in a sterile cup and stored at 4°C while awaiting transport to the laboratory. Women were asked to provide a self-collected vaginal sample, obtained using a Dacron applicator (Qiagen female swab specimen collection kit) and stored at room temperature while awaiting transport to the laboratory. The penile swab was collected from participating men by a clinical study team member and penile cells were collected using two Dacron HC (Qiagen) swabs. One swab sampled the coronal sulcus and glans penis, while the other sampled the penile shaft. Both were combined into one collection vial and stored at room temperature (15). Initial processing was performed in South Africa including centrifugation at 2000 rpm at 4°C for 10 min, 2 ml supernatant saved, followed by re-suspension of the pellet in 10 ml PBS and centrifuged at 2000 rpm at 4°C for 10 min and pellet re-suspended in 3 ml PBS and split into two aliquots. All samples were stored locally at −80°C until shipment to Baltimore lab for DNA purification, amplification and HPV DNA detected by line blot hybridization, as previously described (16, 17). Samples were tested for 37 types of HPV DNA; oncogenic HPV subtypes were defined as 16/18/31/33/35/39/45/51/52/56/58/59/68/73/82 (18–20).

All study participants were asked to complete a self-administered risk factor questionnaire. Individuals from the same couple were asked to fill out the survey in separate rooms to ensure privacy of responses. The questionnaire included questions about risk factors for oral HPV infection including demographics, recent, and lifetime sexual behavior including performing oral sex, alcohol, tobacco, and drug use. Ever regular tobacco use was asked as: “Have you ever smoked cigarettes regularly?” with response options of: yes/no. Ever regular alcohol use was asked similarly. Responses to many of the survey questions were categorical, such as number of oral sexual partners, which was asked as: “In your lifetime, how many different people have you performed oral sex on?” with response options of: 0, 1, 2–5, 6–10, >10.

All study participants were tested for HIV as part of their clinical care and if HIV-positive CD4 cell count by the Zazi clinic as part of their clinical visit. Women with oncogenic vaginal HPV infection were referred for Pap smears.

### Statistical analysis

Characteristics of participants were compared by gender and by HIV status using chi-squared for categorical and test of medians for continuous variables. Chi-squared was used to compare oral and genital HPV prevalence by HIV-status. Fischers exact test was used is comparisons of any group with five or fewer subjects. McNemar’s test was used to compare oral and genital HPV prevalence among the paired male and female couples. Agreement in HPV infections between partners was considered separately by site (oral–oral, genital-oral, and genital–genital) and was described as the proportion of couples who had one or more type-specific concordant infections overall and when limited to subgroups who had one type of infection present. All statistical analyses were performed using STATA version 11.0 (College Station, TX, USA). There were 4/38 couples recruited in which one of the partners opted out of providing a genital sample; therefore all analyses were restricted to the 34 couples with complete oral and genital HPV data. Results were similar when these individuals were not excluded (results not shown).

## Results

This pilot study included 13 individuals who tested positive for HIV infection, as well as 55 people testing HIV-negative, called HIV-uninfected (Table 1). HIV concordance was seen in 2 couples, 9 couples were HIV discordant, and 23 couples were HIV-uninfected. The median CD4 count among HIV-positive individuals was 437 cells/mm^3^ [Interquartile range (IQR): 161–575 cells/mm^3^]. Median age was 31 years (IQR: 28–36). Women were more likely than men to test HIV positive (29 vs. 9%, *p* = 0.03) and women were significantly younger and less likely to use tobacco or alcohol than men in the study (Table 1). Most men 27/33 (82%) and women 26/31 (84%) reported ever performing oral sex. Median number of lifetime sexual partners was 2–5 (IQR: 2–5, 6–10), while median number of lifetime oral sex partners was 1 (IQR: 1, 2–5).

**Table 1:** **Characteristics of 34 couples attending Zazi Clinic for HIV counseling and testing, stratified by gender**.

	All (*N* = 68)	Female (*N* = 34)	Male (*N* = 34)	*p*-Value*
		
		*N* (%)		
Age in years(IQR)	31 (28–36)	30 (24–32.5)	33 (29–37)	0.021
**HIV-STATUS**				
HIV-uninfected	55 (80.9)	24 (70.6)	31 (91.2)	0.031
HIV-infected	13 (19.1)	10 (29.4)	3 (8.8)	
Median CD4 cell count among HIV+ (IQR)^a^	437 (161–575)	460 (272–566)	76 (9–584)	0.307
**EVER REGULAR TOBACCO USE**				
No	45 (66.2)	28 (82.4)	17 (50.0)	0.001
Yes	21 (30.9)	4 (11.8)	17 (50.0)	
Unknown	2 (2.9)	2 (5.9)		
**EVER REGULAR ALCOHOL USE**				
No	29 (42.7)	18 (52.9)	11 (32.4)	0.039
Yes	31 (45.6)	11 (32.4)	20 (58.8)	
Unknown	8 (11.8)	5 (14.7)	3(8.8)	
**EVER PERFORMED ORAL SEX?**				
No	11 (16.2)	5 (14.7)	6 (17.6)	0.828
Yes	53 (77.9)	26 (76.5)	27 (79.4)	
Unknown	4 (5.9)	3 (8.8)	1 (2.9)	
**AGE FIRST TIME PERFORMED ORAL SEX**				
<21	27 (39.7)	15 (44.1)	12 (35.3)	0.653
21–30	22 (32.4)	10 (29.4)	12 (35.3)	
>30	4 (5.9)	1 (2.9)	3 (8.8)	
Never	9 (13.2)	5 (14.7)	4 (11.8)	
Unknown	6 (8.8)	3 (8.8)	3 (8.8)	
**NUMBER OF PARTNERS PERFORMED ORAL SEX ON IN LIFETIME**				
None	11 (16.2)	6 (17.6)	5 (14.7)	0.956
1	21 (30.9)	10 (29.4)	11 (32.4)	
2–5	26 (38.2)	13 (38.2)	13 (38.2)	
6–10	5 (7.4)	2 (5.9)	3 (8.8)	
Unknown	5 (7.4)	3 (8.8)	2 (5.9)	
**NUMBER OF VAGINAL SEX PARTNERS IN LIFETIME**				
None	1 (1.5)	1 (2.9)	0 (0.0)	0.308
1	5 (7.4)	4 (11.8)	1 (2.9)	
2–5	29 (42.7)	17 (50.0)	12 (35.3)	
6–10	9 (13.2)	3 (8.8)	6 (17.7)	
11–15	7 (10.3)	2 (5.9)	5 (14.7)	
16–24	3 (4.4)	2 (5.9)	1 (2.9)	
≥25	1 (1.5)	0 (0.0)	1 (2.9)	
Unknown	13 (19.1)	5 (14.7)	8 (23.5)	

*p Values were calculated using the Mann–Whitney test for continuous variables and chi-squared for all remaining variables and excluded those with unknown values.

*^a^ CD4 cell counts were available for only 11 of the HIV-infected individuals; this included 8 females and 3 males*.

### HPV prevalence

There were a total of 35 different genital HPV types detected amongst the study participants, including HPV 16 and 62 (prevalence each 16.2%), 45 and 52 (each 11.8%), 61 (10.3%), 66 (8.8%), 6, 18, 35, 72, 84, 89 (each 7.4%) as the most common types. For oral HPV the most common infections detected were HPV 62 (prevalence 7.4%), 72 (5.9%), 35, 52, 33, 58 (each 2.9%), and 16, 74, 66 (each 1.5%).

Oral HPV prevalence was significantly lower than genital HPV prevalence (15 vs. 75%, *p* ≤ 0.001) (Table 2) but oral HPV prevalence was similar in women and men (12 vs. 18%, *p* = 0.48), and was higher, but not statistically different, among HIV-infected vs. HIV-uninfected individuals (23 vs.13%, *p* = 0.34). Genital HPV prevalence was significantly higher in women vs. men (91 vs. 59%, *p* = 0.005) and HIV-infected vs. HIV-uninfected (100 vs. 69%, *p* = 0.02). Oncogenic HPV subtypes were detected in 4% of oral, 29% of penile, and 74% of cervical samples, including HPV 16 in 1, 12, and 21% of these samples respectively.

**Table 2:** **Oral and Genital HPV Prevalence by gender and HIV status**.

	*N* (%)					
	Female (*N* = 34)	Male (*N* = 34)	*p*-Value*	HIV-infected (*N* = 13)	HIV-uninfected (*N* = 55)	*p*-Value**
**ORAL HPV**						
Any	4 (11.8)	6 (17.7)	0.48	3 (23.1)	7 (12.7)	0.34
Any oncogenic subtype (16, 18, 31, 33, 35, 39, 45, 51, 52, 56, 58, 59, 68, 73, 82)	1 (2.9)	2 (5.9)	0.56	1 (7.7)	2 (3.6)	0.52
Any vaccine subtype (6, 11, 16, 18)	0 (0)	1 (2.9)	0.32	0 (0)	1 (1.8)	0.62
HPV 16	0 (0)	1 (2.9)	0.32	0 (0)	1 (1.8)	0.62
Multiple subtypes	2 (5.9)	4 (11.8)	0.41	2 (15.4)	4 (7.3)	0.35
**GENITAL HPV**						
Any	31 (91.2)	20 (58.8)	0.005	13 (100.0)	38 (69.1)	0.02
Any oncogenic subtype (16, 18, 31, 33, 35, 39, 45, 51, 52, 56, 58, 59, 68, 73, 82)	25 (73.5)	10 (29.4)	0.0001	10 (76.9)	25 (45.5)	0.04
Any vaccine subtype (6, 11, 16, 18)	13 (38.2)	6 (17.65)	0.02	9 (69.2)	10 (18.2)	<0.0001
HPV 16	7 (20.6)	4 (11.8)	0.18	4 (30.8)	7 (12.7)	0.11
Multiple subtypes	20 (58.8)	7 (20.6)	0.0003	11 (84.6)	16 (29.1)	<0.0001

*p Values comparing male and female partners were calculated using McNemar’s test for the comparison between couples.

**p Values comparing HIV+ and HIV− subjects were calculated using Chi-squared for the comparison between HIV+ and HIV− individuals.

Comparing HIV-infected vs. HIV-uninfected individuals, HIV-infected individuals displayed a statistically significantly higher prevalence of both genital oncogenic HPV subtypes (76.9 vs. 45.5%, *p* = 0.04) and multiple HPV infections (84.6 vs. 29.1%, *p* ≤ 0.0001). For oral samples, there was a trend for higher oncogenic HPV prevalence (7.7 vs. 3.6%, *p* = 0.52) and multiple HPV infections (15.4 vs. 7.3%, *p* = 0.35) in HIV-infected vs. HIV-uninfected individuals, but this finding was not statistically significant.

### Concordant HPV infection

**Figure 1:**
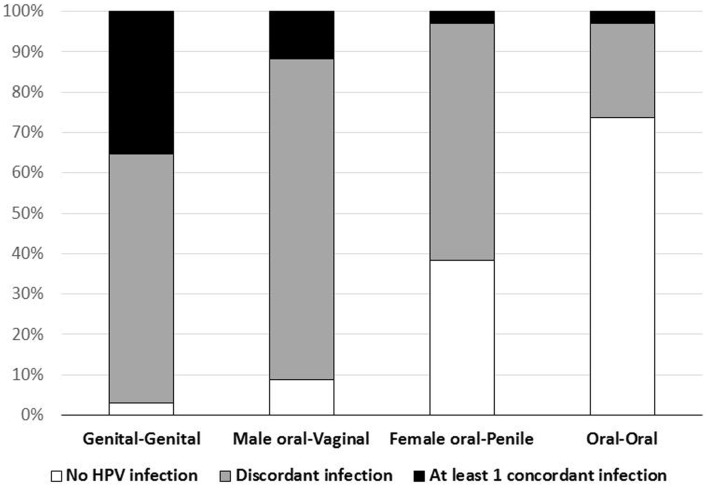
**Frequency of type-specific HPV concordance, by site, among 34 couples**.

Concordant type-specific oral-genital HPV infection was detected in 4/34 (12%) couples (Figure 1). In 3 (75%) of these four couples the partner with oral HPV reported ever performing oral sex. Among the four couples with concordant oral-genital HPV infection, two couples had concordant HPV 62 infection, one couple had concordant HPV 16 infection, and one couple had multiple concordant subtypes (HPV 33/35/58) (Table 3). There were nine couples where at least one individual had an oral HPV infection, and only one of these couples had concordant oral–oral infection (this couple also had genital–genital and genital-oral concordance with this same HPV type; Table 3). Interestingly, in this couple neither partner reported ever performing oral sex. Among those with oral HPV, men with oral HPV infection (four of six; 67%) were more likely than women with oral HPV infection (one of four; 25%) to have a partner with concordant genital HPV infection (*p* = 0.52).

**Table 3:** **Oral-genital concordance couple characteristics among nine couples with at least one oral HPV infection**.

Couple number	Oral HPV		Genital HPV		HIV status		Ever oral sex	
	Male	Female	Male	Female	Male	Female	Male	Female
1	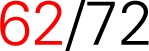	62	62	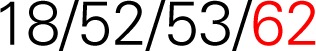	HIV-uninfected	HIV-infected	No	No
2	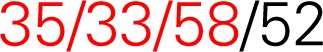	–	6/35/42/45	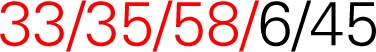	HIV-uninfected	HIV-infected	Yes	Yes
3	16	–	16/66	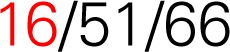	HIV-uninfected	HIV-uninfected	Yes	Yes
4	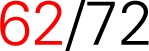	–	–	62	HIV-uninfected	HIV-uninfected	Yes	Yes
5	62/72	–	16/33/35	16/33/35	HIV-uninfected	HIV-uninfected	Yes	No
6	72	–	54	16/54	HIV-uninfected	HIV-uninfected	Yes	Yes
7	–	52/33/35/58	–	62/72	HIV-uninfected	HIV-infected	Yes	Yes
8	–	66	52	66/82	HIV-uninfected	HIV-uninfected	Yes	Yes
9	–	62/74	6/11/33/39/45/64/66/67/83/84	26/45/66/82/83	HIV-infected	HIV-infected	Yes	Yes

*Concordant oral-genital infections shown in red*.

At least one type-specific concordant vaginal-penile HPV infection was observed in 35% of couples (Figure 1). The proportion with concordant vaginal-penile HPV infection varied by HIV status as HIV-uninfected couples displayed 22% concordance, HIV-discordant couples displayed 56% concordance, and HIV-concordant couples displayed 100% concordant vaginal-penile infection (*p* = 0.028).

### Risk factors for prevalent oral HPV infection

In multivariate analysis, oral HPV prevalence was higher, although not statistically significantly different, among HIV-positive individuals (OR: 5.35; 95% CI 0.64–44.69), males (OR: 5.06; 95% CI 0.71–36.21), and those with ≥2 lifetime oral sex partners compared to those with <2 lifetime oral sex partners (OR: 4.24; 95% CI 0.76–23.62), Table 4.

**Table 4:** **Unadjusted and adjusted odds ratios of oral HPV infection (*n* = 68)**.

	Unadjusted OR	95% CI	Adjusted OR	95% CI
Age (per 5 years)	1.66	0.91–3.03	2.24	0.94–5.35
**HIV STATUS**				
HIV-uninfected	1.00	1.00	1.00	1.00
HIV-infected	2.06	0.45–9.35	5.35	0.64–44.69
**GENDER**				
Female	1.00	1.00	1.00	1.00
Male	1.61	0.41–6.30	5.06	0.71–36.21
**NUMBER OF ORAL SEX PARTNERS**				
<2 Lifetime oral sex partners	1.00	1.00	1.00	1.00
≥2 Lifetime oral sex partners	5.22	1.01–26.95	4.24	0.76–23.62

## Discussion

This is one of the first studies to explore oral-genital HPV concordance in couples. We found that 12% of couples displayed oral-genital concordance and among these couples the majority had male oral HPV infection concordant with female vaginal HPV infection. This is consistent with higher likelihood of vaginal-to-oral transmission as compared to penile-to-oral transmission, and may help explain previously reported findings of a lack of significant association between oral-penile contact and incident oral HPV infections amongst women (21). Oral–oral HPV concordance was rare in our study, even among HIV-infected individuals, consistent with no or low salivary transmission.

Our study demonstrated oral HPV prevalence of 15%, consistent with previous studies from South Africa, which showed an oral HPV prevalence of 20–25% (22, 23). Risk factors for oral HPV infection were similar to those reported in other studies, including performing oral sex, male gender, and HIV-infection (4, 24).

Vaginal HPV was detected in >90% of females in our study and the majority of these individuals were found to have oncogenic HPV subtypes. Direct comparisons to previously reported estimates of vaginal HPV amongst women in South Africa are difficult as previous studies report HPV prevalence based on degree of dysplasia present, however population based estimates previously reported for any HPV subtype in women with normal cytology were roughly 21% (25, 26) and ranged from 3.6 to 63% for high-risk subtypes 16 and 18 depending on degree of dysplasia present (22, 25–29). Additionally, HPV prevalence amongst HIV-positive women in South Africa approaches 80% (11). Our overall reported vaginal HPV prevalence exceeds these previously reported estimates, although our sample size was small. When looking solely at oncogenic subtypes (i.e., high-risk) or HPV 16 alone, our findings appear similar (17, 18, 20).

Penile HPV was detected in 59% of males and 29% were found to have oncogenic HPV subtypes. The prevalence of high-risk HPV subtypes amongst males in South Africa has been previously reported as 15% for circumcised males and 22% for uncircumcised males (30). In our study, we did not categorize men by circumcision status, and our reported prevalence is moderately higher than these previous studies. There was a strong correlation between HIV status and both genital HPV prevalence and genital–genital HPV concordance between couples, which is consistent with previous studies (31). For oral HPV there was a trend toward higher prevalence amongst HIV-positive individuals, but this finding was not statistically significant.

While this study was cross sectional, the agreement in oral-genital HPV infection between couples is consistent with transmission by oral sex. Additionally, in three out of the four couples with concordant oral-genital HPV infection, the partner with oral HPV reported having performed oral sex, a finding consistent with previous reports of an association between oral HPV prevalence and reported oral sex (32). The remaining couple displayed both oral–oral and oral-genital HPV concordance, but reported no oral sex. Possible modes of transmission in this couple could include deep kissing or auto-inoculation, as both of these modes of transmission have previously been suggested, although not clearly demonstrated (33, 34). However, oral–oral HPV concordance was low suggesting HPV may not be transmitted by deep kissing, or may be rare. It is noteworthy that oral-genital concordance was substantially lower than genital–genital concordance, this could be because oral sex is less frequent than vaginal sex and/or because per-act transmissibility could be different for oral vs. genital sex.

This study had several limitations. The small sample size limited the analysis of risk factors for infection. Because data was collected in categories exact medians could not be recorded. Additionally, our study provides cross-sectional data only so could not demonstrate transmission over time from one partner to another. However this study provides some of the first estimates of oral–oral and oral-genital HPV concordance in couples in Africa.

This research demonstrates high prevalence of genital HPV infection and moderate prevalence of oral HPV infection in a South African population with a modest number of sexual partners. The HPV vaccine has recently been shown to be effective in preventing oral, as well as cervical and anal HPV infection (35), and this data underscores the importance of preventative HPV vaccination in South Africa. The concordant oral-genital HPV infection detected in these couples supports the transmission of HPV infection to the mouth by oral sex. Given higher HPV prevalence, persistence, and HPV-associated cancer risk among HIV-infected individuals (8), the prevalence of oncogenic oral HPV infection in this population is of note. HPV-associated oropharyngeal cancer incidence has increased in the U.S. and some European countries over the past several decades (3). Given the high HIV prevalence in South Africa and other countries in the region, as individuals with HIV live longer due to effective ART, it is not known whether a similar increase in HPV-associated oropharyngeal cancer will be observed in this region.

## Conflict of Interest Statement

Gypsyamber D’Souza has research support for Merck Inc. The other co-authors declare that the research was conducted in the absence of any commercial or financial relationships that could be construed as a potential conflict of interest.
